# Association of MMP-8 rs11225395 Polymorphism with the Susceptibility of Peri-Implantitis

**DOI:** 10.3290/j.ohpd.c_2153

**Published:** 2025-08-05

**Authors:** Huadan Jin, Yihan Fu, Dan Zhao, Liye Wang, Yaoyan Wang, Rongqing Hu, Renjie Fu

**Affiliations:** a Huadan Jin Oral Implantology Department, Jinhua Jinhao Dental Clinic, Jinhua 321000, China. Conceptualisation, data curation, methodology, software, visualisation, writing – original draft. Huadan Jin and Yihan Fu contributed equally to this work.; b Yihan Fu General Dentist, Smilebuilderz, PA 17601, USA. Conceptualisation, data curation, methodology, software, visualisation, writing – original draft. Huadan Jin and Yihan Fu contributed equally to this work.; c Dan Zhao General Dentistry, Wuxi Stomatology Hospital, Wuxi 214000, China. Conceptualisation, data curation, formal analysis, methodology, resources, software; d Liye Wang General Dentistry, Wuxi Stomatology Hospital, Wuxi 214000, China. Conceptualisation, data curation, formal analysis, investigation, methodology, resources, software, validation.; e Yaoyan Wang Endodontics, Wuxi Stomatology Hospital, Wuxi 214000, China. Conceptualisation, data curation, formal analysis, investigation, resources, validation.; f Rongqing Hu General Dentistry, Wuxi Stomatology Hospital, Wuxi 214000, China. Conceptualisation, data curation, investigation, project administration, supervision, validation.; g Renjie Fu Department of Stomatology, The Tongxiang First People’s Hospital, Tongxiang 314500, China. Conceptualisation, data curation, funding acquisition, project administration, supervision, writing – review and editing.

**Keywords:** MMP-8, Rs11225395, polymorphism, peri-implantitis

## Abstract

**Purpose:**

Peri-implantitis (PI) is the primary cause of implant failure, and genetic susceptibility significantly influences its development. This study investigated the association between the MMP-8 rs11225395 polymorphism and PI in the Chinese Han population.

**Materials and Methods:**

In this study, 140 Chinese Han patients diagnosed with PI and 156 healthy implant controls were included. Polymerase chain reaction (PCR) was employed to detect the MMP-8 rs11225395 polymorphism, and the Hardy–Weinberg equilibrium test was conducted to assess the representativeness of the samples. Additionally, enzyme-linked immunosorbent assay (ELISA) was utilised to quantify the expression levels of MMP-8, and logistic regression analysis was performed to identify independent risk factors for PI disease.

**Results:**

There were statistically significant differences in the genotype and allele distributions of the MMP-8 rs11225395 locus between PI patients and the control group. Rs11225395 T allele was significantly associated with an increased risk of PI, particularly for individuals with the TC/TT genotype who exhibited higher susceptibility to the disease. Periodontal status indicators differed markedly among PI patients with different genotypes. Factors such as plaque index, brushing daily, probing pocket depth (PPD), clinical attachment loss (CAL), and MMP-8 rs11225395 polymorphism played crucial roles in PI risk. Additionally, MMP-8 expressions were upregulated in PI patients. Specifically, at the rs11225395 locus, individuals with the TC/TT genotype showed a significantly higher relative expression level of MMP-8.

**Conclusion:**

The MMP-8 rs11225395 polymorphism was significantly associated with genetic susceptibility to PI.

Peri-implantitis (PI) is an inflammatory condition affecting the soft and hard tissues surrounding dental implants and represents one of the most common complications associated with dental implants.^3^ Studies have demonstrated that several factors, including poor oral hygiene practices, implant material properties, design and surface characteristics, bone augmentation surgical techniques, biological material applications, microbial characteristics, improper implant surgical procedures, and local susceptibility factors such as implant positioning and prosthetic design, may contribute to the onset and progression of PI.^4^ Several clinical measures have been developed for the routine assessment of periodontal and peri-implant tissues, including periodontal and peri-implant probing, bleeding on probing, intraoral radiography, biomarker analysis, and microbiological testing.^20,22,24^ In iatrogenic PI, the surge in biological complications has led to increased patient dissatisfaction in terms of time, cost, aesthetics, and maintenance.^8^ The development of PI can have a significant negative impact on the long-term success of dental implants.^14,15^ Progressive bone tissue destruction compromises the stability of the implant, potentially leading to loosening and eventual loss, thereby undermining the effectiveness of implant restoration^9^. It is challenging to achieve a complete resolution of peri-implantitis.^23^ Research shows that genetic polymorphisms have emerged as critical factors in the pathogenesis of PI.^16^ A deeper understanding of genetic susceptibility to PI can provide a crucial theoretical foundation for developing personalised diagnostic and therapeutic strategies for this condition.

Matrix metalloproteinases (MMPs) play a pivotal role in various tissue-destructive inflammatory processes. In PI, MMP-8 and its genetic variations are considered critical factors, not only contributing to pathogenesis but also holding potential diagnostic value^6^. Compared to healthy implants, the concentration of MMP-8 in the peri-implant crevicular fluid (PICF) of PI-diagnosed implants is significantly higher.^13^ Furthermore, the MMP-8 single-nucleotide polymorphism (SNP) has been shown to have significant associations with various diseases. Specifically, among Taiwanese individuals with hypertension, those carrying the MMP-8 rs11225395 CT or TT genotype exhibit a higher risk of renal cell carcinoma (RCC) compared to those with the wild-type CC genotype.^19^ Additionally, the MMP-8 rs11225395 SNP is identified as a risk factor for diabetic cardiomyopathy (DC).^25^ However, the mechanisms underlying the role of the MMP-8 rs11225395 gene polymorphism in the pathogenesis of PI remain unclear.

This study primarily investigated the correlation between the MMP-8 rs11225395 polymorphism and PI in Chinese Han patients. This research seeks to elucidate the role of MMP-8 rs11225395 polymorphism in PI and provide novel insights for its treatment.

## MATERIALS AND METHODS

### Study Objects

Post hoc power analysis was run using GPower 3.1 software to obtain the sample power. Input parameters for two-tailed correlational points by serial were: effect size of 0.5; alpha 0.05; and 140 and 156 samples. Based on the above-mentioned assumptions, the statistical power (1-β err prob) was 0.98. A total of 140 Chinese Han patients diagnosed with PI and 156 healthy implant controls from the author’s institution were enrolled. All subjects met the corresponding inclusion and exclusion criteria. The inclusion criteria were as follows: patients must meet the diagnostic criteria for PI, characterised by redness and swelling of the peri-implant mucosa, bleeding on probing, and a probing depth exceeding the normal range (>4 mm or 5 mm, with or without purulent discharge). Imaging examinations (eg, radiographic films) must show peri-implant bone resorption. Additionally, the implant must have achieved osseointegration and been stable before the onset of PI. Patients must have at least one implant in the oral cavity and be able to understand and consent to participate in the research or treatment plan, including regular follow-up visits and acceptance of relevant examinations and treatments. The exclusion criteria included: the presence of other severe oral diseases; implant failure due to quality issues, design defects, or improper placement; receipt of PI-related treatments within the past 3–6 months; presence of severe systemic diseases such as uncontrolled cardiovascular, haematological, or psychiatric conditions; individuals diagnosed with type 2 diabetes; allergic reactions to medications or materials used in the study or treatment; and incomplete medical records or lack of cooperation during examinations. Table 1 summarises the general clinical information of the enrolled subjects.

**Table 1:** Characteristics of controls and PI

Items	Subjects (n = 296)	P
Controls group (n = 156)	PI group (n = 140)
Age (years)	43.08 ± 5.21	43.17 ± 4.43	0.878
Geder (male/female)	80/76	65/75	0.404
Smoking (n/%)			0.024
Yes	80 (51.28)	90 (64.29)	
No	76 (48.72)	50 (35.71)	
Drinking alcohol (n/%)			0.159
Yes	83 (53.21)	63 (45.00)	
No	73 (46.79)	77 (55.00)	
Periodontitis (n/%)			
Yes	70 (44.87)	77 (55.00)	0.082
No	86 (55.13)	63 (45.00)	
Tooth loss reason (n/%)			0.465
Periodontitis	68 (43.59)	71 (50.71)	
Deep caries	1 (0.64)	1 (0.72)	
Trauma	87 (55.77)	68 (48.57)	
Peri-implant phenotype (n/%)			0.686
Thin	85 (54.49)	73 (52.14)	
Thick	71 (45.51)	67 (47.86)	
Position (n/%)			0.394
Anterior region	100 (64.10)	83 (59.29)	
Posterior region	56 (35.90)	57 (40.71)	
Brushing daily (n/%)			0.01
1–3 times	131 (83.97)	131 (93.57)	
More than three times	25 (16.03)	9 (6.43)	
Mouth washing daily (n/%)			0.435
Yes	46 (29.49)	51 (36.43)	
No	35 (22.44)	27 (19.29)	
Infrequent	75 (48.07)	62 (44.28)	
Periodontal status of subjects			
Gingival index	0.58 ± 0.20	2.40 ± 0.28	<0.001
Plaque index	0.85 ± 0.20	2.26 ± 0.34	<0.001
Calculus index	0.24 ± 0.15	0.56 ± 0.22	<0.001
PPD (mm)	1.87 ± 0.43	3.88 ± 0.1.85	<0.001
CAL (mm)	1.35 ± 0.19	2.40 ± 1.53	<0.001
Annotation: PI, peri-implantitis; PPD, peri-implant pocket depth; CAL, clinical attachment level. Data are expressed as mean ± standard deviations (SD) or n/%.

The peri-implant probing depth (PPD) was defined as the distance between the dental implant and the gingival margin, measured using a manual probe from the crest of the gingival margin to the base of the gingival sulcus1. The amount of bone loss around the dental implant, known as crystal bone loss (CBL), was quantified by measuring the straight-line distance from 2 mm apical to the implant-abutment junction to the most coronal point of the alveolar bone.^2^

This study received approval from the Ethics Committee of the author’s institution, and all participants provided informed consent. Additionally, all procedures were conducted in strict accordance with the Helsinki Declaration.

### Genotyping of SNPs

Before DNA extraction, patients were instructed to abstain from eating or drinking for 30 min and rinse their mouths with water. Sterile cotton swabs were used to collect oral mucosa samples, which were immediately placed in sterile tubes. Genomic DNA was extracted using an oral swab genomic DNA extraction kit. The purity of the extracted DNA was assessed by measuring the A260/A280 ratio. PCR amplification was performed using the extracted genomic DNA as a template. Following purification, real-time PCR genotyping of SNPs rs11225395 was conducted on the PCR products using the ABI3730 genetic analyser. The MMP-8 rs11225395 primer sequence was (forward: 5’ – TTCACATAGCCT TGGGAGG – 3’, and reverse: 5’ – TGGGAGACTACCATGCAGATC – 3’). The conditions for PCR amplification were as follows: initial denaturation was performed at 95°C for 15 min, followed by 35 cycles with denaturation at 95°C for 30 s, annealing at 58°C for 30 s, and extension at 72°C for 30 s. A final extension was carried out at 72°C for 5 min. Approximately 10% of the samples were randomly selected for repeat testing, and the results demonstrated 100% consistency. The PCR products were subsequently verified by agarose gel electrophoresis. Finally, raw data were collected and analysed using GeneMapper 4.1 software.

### MMP-8 Concentration was Measured by Enzyme-Linked Immunosorbent Assay (ELISA)

MMP-8 level was estimated by enzyme-linked immunosorbent assay using Quantikine human total MMP-8 immunoassay kit (R&D Systems, MN, USA). Special filter strips or capillary tubes were inserted into the gingival sulcus surrounding the implant to collect the gingival crevicular fluid. The required samples were centrifuged and subsequently incubated at 4°C for 15 min. Detection antibodies were added, followed by gentle mixing, and the samples were incubated at room temperature for 16 to 24 h. The microtiter plates were washed three times, after which the control group and the PI group were added to their respective wells in triplicate. Following incubation, the plates were washed again and then incubated with the conjugate at room temperature for 1 h, followed by incubation with the substrate for 15 min. The reaction was terminated using a stop solution, and the absorbance was measured using a fully automatic microplate reader. The concentration of MMP-8 was quantified in units of ng/mL.

### Statistical Analysis

SPSS 26.0 was utilised for statistical processing and analysis of data. Continuous variables were analysed using t-tests, while categorical variables were assessed via chi-square tests. The Hardy–Weinberg equilibrium test was conducted to evaluate the representativeness of the samples to the population. Logistic regression analysis was employed to assess the association between the MMP-8 rs11225395 polymorphism and susceptibility to PI. ELISA was used to measure the concentration of MMP-8. Count data were presented as cases (%), and continuous data were reported as mean ± standard deviation. P <0.05 was considered statistically significant.

## RESULTS

### Clinical Features of Study Subjects

This study enrolled 140 patients with PI and 156 healthy controls and compared the general clinical information and various characteristics between the two groups (Table 1). No significant differences were observed between the two groups in terms of age, gender, drinking habits, prevalence of periodontitis, causes of tooth loss, peri-implant phenotypes, positions, and mouth rinsing practices (P >0.05). However, statistically significant differences were noted in periodontal status-related characteristics between the control group and the PI group. Specifically, smoking, daily tooth brushing, the gingival index, plaque index, calculus index, PPD, and CAL were all sstatistically significantly higher in the PI group (P <0.05). These findings suggested that these periodontal indicators were specific and sensitive markers for PI and could serve as key parameters for its diagnosis, treatment, and research.

#### Association between PI Risk and MMP-8 rs11225395 polymorphisms

We conducted an in-depth analysis of the correlation between the MMP-8 rs11225395 SNP and PI (Table 2). The results demonstrated a statistically significant association between the MMP-8 rs11225395 SNP and PI. In the codominant model, the genotype frequencies in the control group were as follows: CC (35.90%), TC (44.87%), and TT (19.23%). In contrast, the PI group exhibited the following genotype frequencies: CC (21.43%), TC (55.71%), and TT (22.86%). Regarding allele frequencies, the C allele was present at 58.33% in the control group and 49.29% in the PI group, while the T allele was observed at 41.67% in the control group and 50.71% in the PI group. Statistical analysis revealed that the higher frequency of the T allele in the PI group was statistically significantly associated with an increased risk of developing PI (P <0.001, OR = 1.441). Additionally, there were notable differences in the distribution of CC, TC, and TT genotypes between the PI and control groups, with individuals carrying the TC or TT genotypes being more susceptible to PI (P = 0.008, OR = 2.080 for TC; P = 0.042, OR = 1.991 for TT).

**Table 2:** Correlation of MMP-8 rs11225395 SNP in PI patients

SNPs	Control group n = 156 (%)	PI group n = 140 (%)	_χ_2	P	OR (95% CI)
Codominant model					
CC	56 (35.90)	30 (21.43)	–	–	1.0
TC	70 (44.87)	78 (55.71)	6.949	0.008	2.080 (1.202–3.600)
TT	30 (19.23)	32 (22.86)	4.142	0.042	1.991 (1.022–3.879)
Dominant model					
CC	56 (35.90)	30 (21.43)	–	–	1.0
TC/TT	100 (64.10)	110 (78.57)	7.494	0.006	2.053 (1.221–3.452)
Recessive model					
CC/TC	126 (80.77)	108 (77.14)	–	–	1.0
TT	30 (19.23)	32 (22.86)	0.586	0.444	1.244 (0.710–2.180)
Alleles					
C	182 (58.33)	138 (49.29)	–	–	1.0
T	130 (41.67)	142 (50.71)	4.864	0.027	1.441 (1.041–1.994)
*P* ^HWE^	0.337				
Notes: PI, peri-implantitis; SNP, single nucleotide polymorphism; OR, odds ratio; CI, confidence interval.

In the dominant model, 35.90% of individuals carried the CC genotype, while 64.10% carried the TC or TT genotypes. Correlation analysis revealed that individuals with the TC or TT genotypes were statistically significantly more susceptible to PI (P = 0.006, OR = 2.053). In the recessive model, no statistically significant difference was observed in PI occurrence between individuals with the CC or TC genotypes and those with the TT genotype (P> 0.05). Furthermore, the Hardy–Weinberg equilibrium test value (PHWE = 0.337) indicated that the gene frequency distribution in the population was stable. Collectively, these findings suggested that the MMP-8 rs11225395 SNP may play a crucial role in the pathogenesis or susceptibility to PI.

#### Periodontal status in patients with MMP-8 rs11225395 polymorphism

We further investigated the periodontal status of patients with the MMP-8 rs11225395 polymorphism (Table 3). Among the PI patients, they were categorised into the CC genotype group (n = 30) and the TC/TT genotype group (n = 110). Statistically significant differences were observed in periodontal status indicators, including the gingival index, plaque index, calculus index, PPD, and CAL, between the two groups (P <0.001). These findings suggested that the MMP-8 rs11225395 polymorphism may play a critical role in the pathological changes of periodontal tissues associated with PI, indicating that this genetic variant statistically significantly influences the periodontal status of PI patients.

**Table 3:** Periodontal status in patients with rs11225395 gene polymorphism

Items	CC (n = 30)	TC/TT (n = 110)	P value
Gingival index	2.18 ± 0.21	2.47 ± 0.27	<0.001
Plaque index	1.95 ± 0.26	2.35 ± 0.31	<0.001
Calculus index	0.40 ± 0.16	0.61 ± 0.21	<0.001
PPD (mm)	2.56 ± 1.59	4.26 ± 1.75	<0.001
CAL (mm)	1.45 ± 0.81	2.66 ± 1.59	<0.001
Note: Data were expressed as mean and standard deviation (SD). PI, peri-implantitis; PPD, peri-implant pocket depth; CAL, clinical attachment level.

#### Rs11225395 polymorphisms were independent risk factors for the development of PI

To further evaluate the relationship between MMP-8 rs11225395 and the risk of PI, we conducted a logistic regression analysis (Table 4). Gingival index, smoking, and calculus index were not statistically significantly associated with the risk of PI (P >0.05). In contrast, plaque index, brushing daily, PPD, CAL, and the rs11225395 polymorphism were statistically significantly associated with an increased risk of PI (P <0.05). These findings suggested that when assessing the risk of PI, factors such as oral hygiene practices (brushing daily), plaque index, PPD, CAL, and the MMP-8 rs11225395 gene polymorphism played crucial roles in disease onset. Specifically, plaque index, poor oral hygiene habits (not rinsing the mouth daily), abnormal PPD and CAL values indicative of tissue destruction around the implant, and variations in MMP-8 rs11225395 were all statistically significantly associated with an elevated risk of PI. Therefore, in clinical practice and research, it is essential to focus on these critically influencing factors to more accurately prevent and manage PI.

**Table 4:** Logistic regression analysis of risk factors for peri-implantitis

Variables	Logistic regression analysis
OR	95% CI	P value
Smoking	1.180	0.747–1.864	0.477
Brushing daily	0.478	0.259–0.882	0.009
Gingival index	1.235	0.782–1.951	0.365
Plaque index	1.853	1.167–2.942	0.009
Calculus index	1.297	0.821–2.051	0.265
PPD (mm)	1.607	1.014–2.547	0.043
CAL (mm)	1.868	1.168–2.987	0.009
Rs11225395	1.969	1.175–3.298	0.010
Notes:PPD, peri-implant pocket depth; CAL, clinical attachment level; OR,odds ratio; CI,confidence interval.

#### The expression of MMP-8 was upregulated in patients with PI

**Fig 1a and b:**
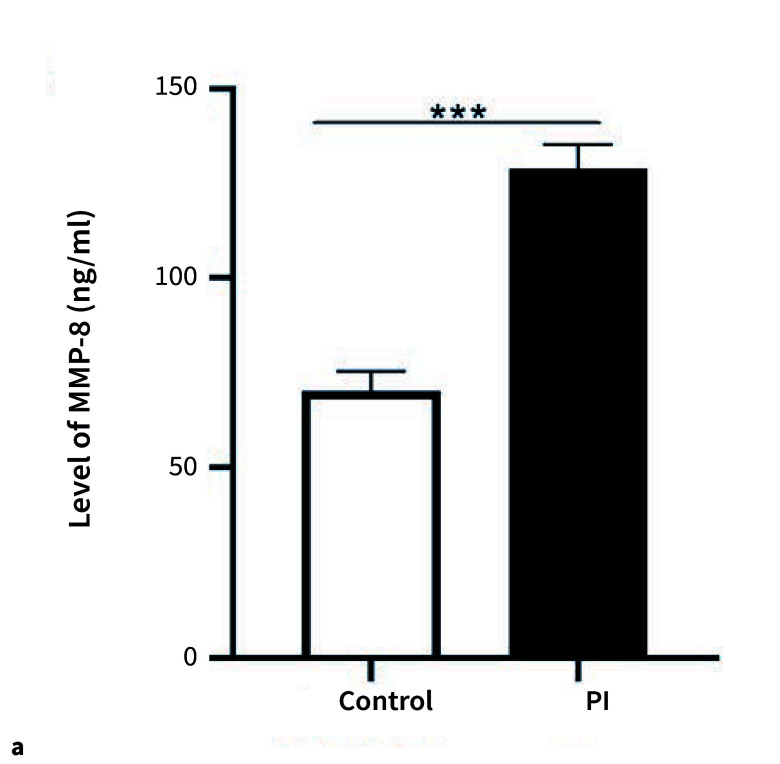

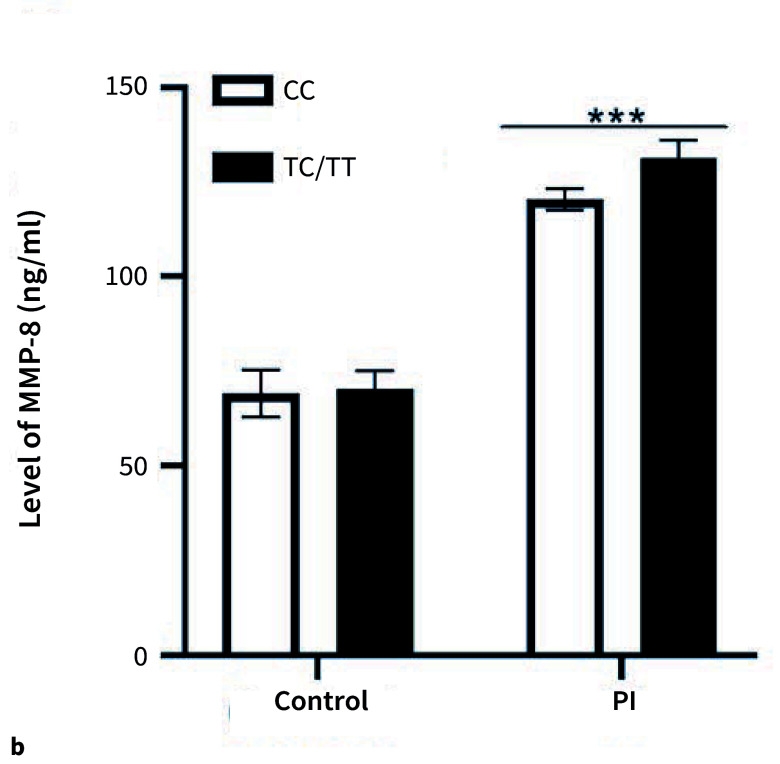
Relative MMP-8 expression in the PI patients. (a) The expression of MMP-8 was upregulated in patients with PI. (b) In patients with PI, individuals carrying the TC/TT genotype exhibited higher levels of MMP-8. *** means P <0.001.

To further elucidate the regulatory mechanism of MMP-8 in PI, we conducted a comprehensive study on the expression levels of MMP-8. The results demonstrated that MMP-8 expression was statistically significantly elevated in PI patients (Fig 1a). This suggested that MMP-8 may be involved in the pathophysiological processes associated with PI, such as degrading extracellular matrix components surrounding the implant, disrupting normal tissue structure, and promoting inflammation, soft tissue destruction, and alveolar bone resorption, thereby serving as a critical factor in the pathogenesis of PI. Additionally, our findings revealed genotype-specific differences in MMP-8 expression at the rs11225395 locus (CC, TC/TT) (Fig 1b). In the PI group, individuals carrying the TC/TT genotype exhibited statistically significantly higher MMP-8 expression levels than those with the CC genotype. This indicated that genetic polymorphisms at this locus can modulate MMP-8 expression. Different genotypes may influence gene transcription, translation, or the activity of regulatory elements, leading to varied MMP-8 expression levels and impacting susceptibility and severity of PI. In conclusion, MMP-8 likely played a crucial role in the onset and progression of PI.

## DISCUSSION

PI is a pathological condition that affects the tissues surrounding dental implants, characterised by inflammation of the peri-implant mucosa and progressive bone loss.^12^ During implant surgery, an excessively large incision of the gingival tissue or improper healing may result in a gap between the implant and the gingiva. This gap can allow oral bacteria to come into direct contact with the implant surface. Furthermore, if the prosthetic design does not conform to the requirements of oral anatomy and physiological functions, it may compromise the efficiency of oral self-cleaning mechanisms.^5,21^ The immune response to bacterial invasion is influenced not only by genetic factors but also by environmental factors. Epigenetic modifications have been demonstrated to play a significant role in the progression of various diseases, including PI inflammation.^18,26^ Research indicates that MMP-8 and its genetic variants are key factors in PI, not only contributing to disease development but also offering potential diagnostic value6. The MMP-8 rs11225395 polymorphism may serve as a potential biomarker for predicting susceptibility to colorectal cancer.^27^ Therefore, we hypothesised that the MMP-8 rs11225395 polymorphism may be associated with PI inflammation.

This study enrolled 140 patients with PI and 156 healthy controls. The results showed that significant differences were observed in the gingival index, plaque index, calculus index, PPD, and CAL. These periodontal indicators serve as key bases for diagnosis, treatment, and research. Gene polymorphisms can influence gene expression and function.^7,17^ A statistically significant association was found between the MMP-8 rs11225395 SNP and PI. Specifically, the frequency of the T allele was notably higher in the PI group, indicating an increased risk of developing PI. Additionally, individuals carrying the TC or TT genotypes were more susceptible to PI. This conclusion was supported by previous studies. For instance, research has shown that in the Taiwanese population, hypertensive individuals carrying the MMP-8 rs11225395 CT or TT genotype have a higher risk of RCC compared to those with the wild-type CC genotype.^19^ Furthermore, the T allele and T allele genotypes (CT, TT) are more frequently observed in generalised aggressive periodontitis (GAgP) cases; compared to the wild C allele and CC genotype, the risk of GAgP is 6.76 times higher.^10^ In summary, our study confirmed that the MMP-8 rs11225395 SNP is statistically significantly associated with PI.

Peri-implantitis is an inflammatory condition caused by plaque and is associated with daily oral hygiene habits.^4,14^ Our results indicated statistically significant differences were observed in periodontal health indicators, including the gingival index, plaque index, calculus index, PPD, and CAL between genotype groups. Logistic regression analysis revealed that when assessing the risk of PI, factors such as poor oral hygiene practices (eg, not brushing daily), plaque index, elevated PPD and CAL values indicative of tissue destruction around the implant, and variations in the MMP-8 rs11225395 gene all contribute to a higher risk of PI development. This indicated that in clinical practice and research, it was crucial to focus on these critical factors to more accurately prevent and manage the occurrence and development of PI. This also underscored that genetic factors, along with environmental and clinical indicators, collectively contribute to the pathogenesis of PI.

We conducted a comprehensive study on the expression levels of MMP-8 in PI patients compared to a control group. The results demonstrated that MMP-8 expression was statistically significantly elevated in PI patients. This suggested MMP-8’s potential involvement in the pathophysiological processes associated with PI. These findings align with previous studies indicating higher MMP-8 levels in the PICF of PI-diagnosed implants compared to healthy implants^13^ and statistically significantly increased MMP-8 levels in PI 11. Furthermore, our analysis revealed genotype-specific differences in MMP-8 expression at the rs11225395 locus. In the PI group, individuals carrying the TC/TT genotype exhibited statistically significantly higher MMP-8 expression than those with the CC genotype. This indicated that genetic polymorphisms at this locus can regulate MMP-8 expression, potentially affecting gene transcription, translation, or the activity of regulatory elements, leading to differential susceptibility and severity of PI.

In future clinical studies, before implant surgery, an MMP-8 rs11225395 gene polymorphism test should be performed to identify high-risk individuals. For patients carrying risk genotypes, post-implantation care should include enhanced oral hygiene guidance, regular follow-up examinations, and close observation of PI tissue conditions to detect and manage early inflammation promptly, thereby preventing PI onset. When evaluating PI risk in patients, in addition to traditional clinical indicators such as oral hygiene habits, plaque index, PPD, and CAL, the MMP-8 rs11225395 gene polymorphism should also be considered a critical factor. For patients with poor oral hygiene and those carrying risk genotypes, vigilance regarding PI occurrence should be heightened, and monitoring should be intensified. Patients carrying the TC or TT genotypes, which were associated with higher MMP-8 expression levels, may require more aggressive treatment strategies, such as increased frequency of scaling and root planing, as well as the use of antibacterial agents, to control inflammation and minimise MMP-8-induced damage to peri-implant tissues.

Furthermore, this study possesses certain limitations that warrant acknowledgement. Firstly, the sample size was relatively limited, and the ethnic composition lacked diversity. Secondly, the sensitivity of the ELISA method employed for MMP-8 detection exhibited certain constraints. To address these limitations, future studies should aim to expand the sample size and incorporate participants from diverse ethnic backgrounds. This approach will facilitate a more comprehensive understanding of the relationship between MMP-8 gene polymorphism and PI. Additionally, combining the ELISA method with other advanced detection techniques, such as RT-qPCR, could enhance the accuracy of MMP-8 expression level measurements. Such improvements would provide a stronger theoretical foundation for clinical treatment strategies.

In summary, the MMP-8 rs11225395 polymorphism may be related to PI susceptibility. Individuals carrying the TC/TT genotype exhibited statistically significantly higher relative expression levels of MMP-8 compared to those with the CC genotype. Moreover, MMP-8 expression levels were markedly elevated in the PI group relative to the control group, suggesting that this polymorphism may play a role in the pathogenesis of PI via mediating MMP-8 expression. These findings provided valuable insights into the mechanisms underlying PI.
